# High *IER5* Gene Expression Is Associated With Poor Prognosis in Glioma Patients

**DOI:** 10.3389/fcell.2021.679684

**Published:** 2021-06-17

**Authors:** Zijun Wu, Dan Wang, Fanxin Zeng, Yanrong Zhang, Guannan Zhu, Yiqi Ma, Bin Song, Su Lui, Min Wu

**Affiliations:** ^1^Huaxi MR Research Center, Department of Radiology, Functional and Molecular Imaging Key Laboratory of Sichuan Province, West China Hospital, Sichuan University, Chengdu, China; ^2^Department of Clinic Medical Center, Dazhou Central Hospital, Dazhou, China; ^3^Department of Radiology, School of Medicine, Stanford University, Stanford, CA, United States

**Keywords:** *IER5*, glioma, prognosis, bioinformatics analysis, biomarker

## Abstract

**Objective:**

Immediate early response 5 (*IER5*) plays a core role in cell cycle and response to irradiation. However, its role in glioma remains unclear. We aimed to evaluate its prognostic significance in glioma based on The Cancer Genome Atlas data resource.

**Methods:**

The Kruskal–Wallis test, Wilcoxon signed-rank test, and logistic regression were employed to explore the relationship between *IER5* expression and clinicopathological features. Kaplan–Meier and Cox regression analyses were implemented to investigate the relationship of *IER5* with prognosis. A nomogram to estimate the impact of *IER5* on prognosis was created based on the Cox multivariate data. We performed gene set enrichment analysis (GSEA) to determine the key signaling cascades associated with *IER5*. Immunohistochemistry was performed to examine *IER5* expression in a tissue microarray (TMA) of glioma samples.

**Results:**

Immediate early response 5 gene expression was elevated in glioma patients. The level of *IER5* was significantly correlated with WHO grade [OR = 6.71 (4.34–10.68) for G4 vs. G2 and G3], IDH (isocitrate dehydrogenase enzyme) status [OR = 13.35 (8.92–20.46) for wild-type (WT) vs. mutated (Mut)], epidermal growth factor receptor status [OR = 8.42 (4.32–18.43) for Mut vs. WT], age [OR = 0.27 (0.18–0.41) for ≤ 60 years vs. >60 years], and histological type [OR = 7.13 (4.63–11.31] for glioblastoma vs. astrocytoma, oligoastrocytoma, and oligodendroglioma). Univariate analyses revealed that high *IER5* expression was linked to short overall survival (OS) [hazard ratio (HR): 3.747; 95% confidence interval (CI): 2.847–4.933; and *P* < 0.001]. High *IER5* expression was linked to poor OS in multivariate analyses (HR: 2.474; 95% CI: 1.552–3.943; and *P* < 0.001). TMA results showed that high IER5 protein levels were related to short OS (HR: 1.84; 95% CI: 1.10–3.07; and *P* = 0.021) and poor disease-specific survival (HR: 1.82; 95% CI: 1.09–3.04; and *P* = 0.023). GSEA showed that many tumor related pathways were enriched differentially in the *IER5*-high expression group. The C-index and calibration plots of the nomogram showed an effective estimation performance in glioma patients.

**Conclusion:**

Herein, we established that *IER5* plays a critical role in glioma progression and prognosis, which might be an important biomarker for the prognosis of glioma patients.

## Introduction

Glioma is one of the most destructive brain tumors and has high morbidity and mortality rates (Kleihues et al., 2002; Louis et al., 2007). High-grade glioma responds poorly to conventional treatment approaches, e.g., surgical resection, postoperative radiotherapy, as well as chemotherapy (Stupp et al., 2005; Vredenburgh et al., 2007). The prognosis of individuals with high-grade glioma has not been considerably improved in recent years. Although surgery, radiotherapy and chemotherapy have increased the median survival time to 12–15 months (Gusyatiner and Hegi, 2018), the 5-year survival rate of individuals with high-grade glioma is less than 5% (Ostrom et al., 2014b). Glioma is an intricate disease with genome instability resulting from accumulating genetic changes. Many oncogene interactions and molecular networks are related to the genesis and progression of glioma. Identifying the oncogenes involved is crucial for determining treatment strategies and evaluating prognosis. Therefore, it is particularly urgent to find biomarkers that are related to the clinical stage of tumors and can be employed to estimate the prognosis of patients. Although many molecular biomarkers are linked to glioma, such as isocitrate dehydrogenase enzyme 1/2 (IDH1/2) mutation and epidermal growth factor receptor (EGFR) overexpression, their reliability and clinical significance are still controversial and need more research (Thakkar et al., 2014; Alexander and Cloughesy, 2017).

The immediate early response 5 (*IER5*) gene codes for a protein that belongs to the immediate early response (IER) protein family. This protein may have a pivotal role in the modulation of the cell response to mitogenic signals. Specifically, it participates in the modulation of the cell cycle, proliferation, resistance to thermal stress, and cell survival under ionizing irradiation (Nakamura et al., 2011; Ishikawa and Sakurai, 2015; Ishikawa et al., 2015; Kawabata et al., 2015). A study found that the overexpression of *IER5* may help HeLa cervical carcinoma cells recover viability after thermal stress through the refolding of heat-denatured proteins (Ishikawa and Sakurai, 2015). Another study found that the overexpression of *IER5* promotes tumor cell anchorage-independent-growth under stress conditions through HSF1 activation (Asano et al., 2016). However, there are no reports available about the relationship between *IER5* and glioma.

In this research, we used tumor RNA sequencing data obtained from The Cancer Genome Atlas (TCGA) data resource. We explored the gene expression level of *IER5* in glioma and non-malignant brain tissues. Then, we investigated the relationship of *IER5* expression with clinicopathological features. We also analyzed the effect of the *IER5* gene on glioma prognosis through multivariate *Cox* regression analysis. The regression model was based on *IER5* gene expression and clinicopathological characteristics. To predict glioma patient prognosis, we developed a nomogram consisting of the expression level of *IER5* and clinicopathological characteristics. We further grouped samples according to the *IER5* gene expression level, and we compared the transcriptome and expression difference between the high- and low- *IER5* expression groups. We explored IER5 protein levels by quantitative immunohistochemistry (IHC) in a tissue microarray (TMA) of glioma samples from patients with known clinical outcomes. We also validated the prognosis value of *IER5* in three independent datasets in Chinese Glioma Genome Atlas (CGGA). To reveal which genes and functional pathways are highly correlated with the expression of *IER5*, gene ontology (GO) along with Kyoto Encyclopedia of Genes and Genomes (KEGG) analyses, and gene set enrichment analysis (GSEA) were performed. Finally, we explored and discussed the possible mechanism between the expression of *IER5* and the development of tumors by analyzing the relationship of *IER5* expression with tumor immune infiltrates.

Our results may reveal new targets and strategies for glioma diagnosis and treatment. Our data suggest that *IER5* expression can be utilized as a prognostic biomarker for the assessment of disease progression or as a prospective therapeutic target for glioma patients.

## Experimental Procedures

### RNA Sequencing Data and Bioinformatics Analysis

From the TCGA data resource, a total of 670 glioma patients with gene expression data (level 3 HTSeq-FPKM and HTSeq-counts) and clinical data including glioblastoma multiforme and low-grade glioma projects were collected. The normal brain tissue RNA-seq data were collected from GTEx datasets. Afterward, all the RNA-seq data were processed uniformly by the Toil process (Vivian et al., 2017) and converted to transcripts per million reads (TPM) values, and all subsequent analyses were performed using the TPM values. Clinical features of the patients, including age, histological type, gender, 1p/19q codeletion, race, WHO grade, IDH status, primary therapy outcome, EGFR status, and PI3CA status, were collected. Our study satisfies the publication guidelines provided by TCGA. To further validate the prognosis value of *IER5*, three independent datasets in the CGGA were used. The CGGA database includes brain tumors datasets over 2,000 samples from Chinese cohorts^1^. The IER5 expression patterns in cancer cell lines were obtained from Broad Institute Cancer Cell Line Encyclopedia (Barretina et al., 2012). The protein expression of IER5 in U251 cell were obtained from the cell atlas in The Human Protein Atlas (Pontén et al., 2008).

### Differentially Expressed Gene Analysis

The patients were grouped into high- and low- expression groups on the basis of the median value of *IER5* expression. We compared the expression profiles (HTseq-Counts) between the high- and low-*IER5* expression groups to identify differentially expressed gene (DEGs) using the DESeq2 R package (Love et al., 2014). The thresholds values for the DEGs were | log2 fold change (FC) | > 2.0 along with adjusted *P* < 0.01.

### Metascape Analysis

The Metascape resource^2^ is a reliable tool utilized to perform gene annotation, as well as gene list enrichment analysis (Zhou et al., 2019). Herein, we used Metascape to explore the enrichment of *IER5-*linked DEGs categorized by process, as well as pathway. Only the terms with *P* < 0.01, an enrichment factor > 1.5, and a minimum count of 3 were considered to be remarkably different between the groups. The PPI (protein-protein interaction) enrichment analysis in Metascape used the following data resources: BioGrid, OminiPath, and InWeb_IM. Besides, the MCODE (Molecular Complex Detection) algorithm was employed to uncover the densely connected network constituents.

### Gene Set Enrichment Analysis

To identify the remarkable functional and cascade differences between the high- and low- *IER5* groups, we used the R package clusterProfiler (3.14.3) to perform GSEA (Yu et al., 2012). For every analysis, we performed 1000 gene set permutations. Only terms with |NES| > 1, adjusted *P* < 0.05, as well as FDR *q* value < 0.25 were considered to be remarkably differentially enriched between the groups.

### Immune Infiltration Analysis

We employed the ssGSEA (single-sample gene set enrichment analysis) method from the GSVA package^3^ in R (v3.6.2) to perform analysis of the association of immune infiltration with *IER5* expression. We analyzed the invading levels of 24 immune cell types on the basis of the signature genes published in a previous study (Bindea et al., 2013). We employed Spearman correlation analysis to determine the association of *IER5* with immune cells, and the Wilcoxon rank sum test to explore the differences in the invasion of immune cells between the high- and low-*IER5* expression groups.

### Human Glioma Tissue Microarray and Immunohistochemistry

Immunohistochemistry studies of IER5 were performed on glioma samples in a TMA. The human glioma TMA (Product number: HBraG180Su01) was purchased from Shanghai Outdo Biotech Co., Ltd. (Shanghai, China). Ethical approval was granted by ethics committee of Shanghai Outdo Biotech Company. The 180 cases of glioma in this microarray were from Chinese National Human Genetic Resources Sharing Service Platform^4^. And the platform number is 2005DKA21300. In brief, all the samples were incubated with an anti-*IER5* antibody (Invitrogen, PA5-56287, United States; 1:300 dilution) overnight at 4°C. Afterward, the samples were incubated with HRP-labeled secondary antibodies for 1 h at 37°C. Finally, the samples were stained and imaged. EnVision Plus System-HRP (K8002, DAKO, Denmark) was employed, and IER5 was visualized with diaminobenzidine (DAB) as the substrate. The nucleus was stained with Mayer’s hematoxylin counterstain (GT100540, Gene). The assessment of IHC data was carried out by two readers and verified by two independent pathologists blinded to the clinicopathologic information. The staining intensity was defined as: 3 (strong), 2 (moderate), 1 (weak), and 0 (negative). Negative and weak staining intensity was classified as low IER5 expression, whereas strong and moderate staining intensity was classified as high IER5 expression.

### Statistical Analyses

All statistical analyses were conducted using R. Wilcoxon signed rank test and Wilcoxon rank sum test were implemented to investigate the expression of *IER5* in tumor and control samples. The Kruskal–Wallis test, Spearman’s correlation test, Wilcoxon signed rank test, along with Wilcoxon rank sum test were employed to analyze the correlations between clinicopathological features and the expression of *IER5*. Pearson’s χ^2^ test, Fisher’s exact test and univariate logistic regression were implemented to assess the association of clinicopathological variables with the expression of *IER5*. Univariate along with multivariate Cox regression analyses were performed to establish independent variables. In univariate analyses, the significant variables (*P* < 0.1) were then put into the multivariate analysis. The Kaplan–Meier approach with 95% confidence intervals (95% CIs) and the log-rank test were used to construct the survival curves and compare them. In this study, receiver operating characteristic (ROC) assessment was performed with the pROC package (v1.8) to evaluate the effectiveness of the expression level of *IER5* for discriminating glioma from healthy samples (Robin et al., 2011). On the basis of Multivariate Cox regression analysis results, a nomogram was constructed with the R packages rms to establish the individual survival probability of patients with glioma. In all tests, *P* values were two sided, and *P* < 0.05 signified statistical significance.

## Results

### Clinical Characteristics

The clinical characteristics of glioma patients from TCGA consisting of primary therapy outcome, WHO grade, histological type, IDH status, gender, 1p/19q codeletion, age, race, EGFR status, and PIK3CA status were collected. As shown in Table 1, a total of 284 females and 386 males were analyzed in this study. The association analysis illustrated that *IER5* expression was remarkably linked to WHO grade (*P* < 0.001), primary therapy outcome (*P* = 0.018), histological type (*P* < 0.001), 1p/19q codeletion (*P* = 0.002), EGFR status (*P* < 0.001), IDH status (*P* < 0.001), and age (*P* < 0.001). No remarkable association of gene expression with other clinicopathologic features was observed (Table 1).

**TABLE 1:** Characteristics of patients with glioma based on TCGA.

Characters	Level	Low expression of *IER5*	High expression of *IER5*	*P* test
*n*		335	335	
WHO grade (%)	G2	158 (53.7%)	58 (18.2%)	< 0.001
	G3	108 (36.7%)	129 (40.4%)	
	G4	28 (9.5%)	132 (41.4%)	
IDH status (%)	Mut	296 (89.4%)	128 (38.8%)	< 0.001
	WT	35 (10.6%)	202 (61.2%)	
1p/19q codeletion (%)	Codel	102 (30.6%)	66 (19.9%)	0.002
	Non-codel	231 (69.4%)	265 (80.1%)	
Primary therapy outcome (%)	CR	84 (31.7%)	51 (28.5%)	0.018
	PD	49 (18.5%)	54 (30.2%)	
	PR	44 (16.6%)	18 (10.1%)	
	SD	88 (33.2%)	56 (31.3%)	
Gender (%)	Female	141 (42.1%)	143 (42.7%)	0.938
	Male	194 (57.9%)	192 (57.3%)	
Race (%)	Asian	5 (1.5%)	8 (2.4%)	0.554
	Black or African American	14 (4.3%)	18 (5.4%)	
	White	308 (94.2%)	305 (92.1%)	
Histological type (%)	Astrocytoma	111 (33.1%)	81 (24.2%)	< 0.001
	Glioblastoma	28 (8.4%)	132 (39.4%)	
	Oligoastrocytoma	82 (24.5%)	46 (13.7%)	
	Oligodendroglioma	114 (34.0%)	76 (22.7%)	
EGFR status (%)	Mut	9 (2.8%)	64 (19.3%)	< 0.001
	WT	316 (97.2%)	267 (80.7%)	
PIK3CA status (%)	Mut	19 (5.8%)	30 (9.1%)	0.156
	WT	306 (94.2%)	301 (90.9%)	
Age (%)	≤ 60	299 (89.3%)	232 (69.3%)	< 0.001
	>60	36 (10.7%)	103 (30.7%)	

### Relationship of *IER5* Expression With Clinicopathologic Features

**FIGURE 1:**
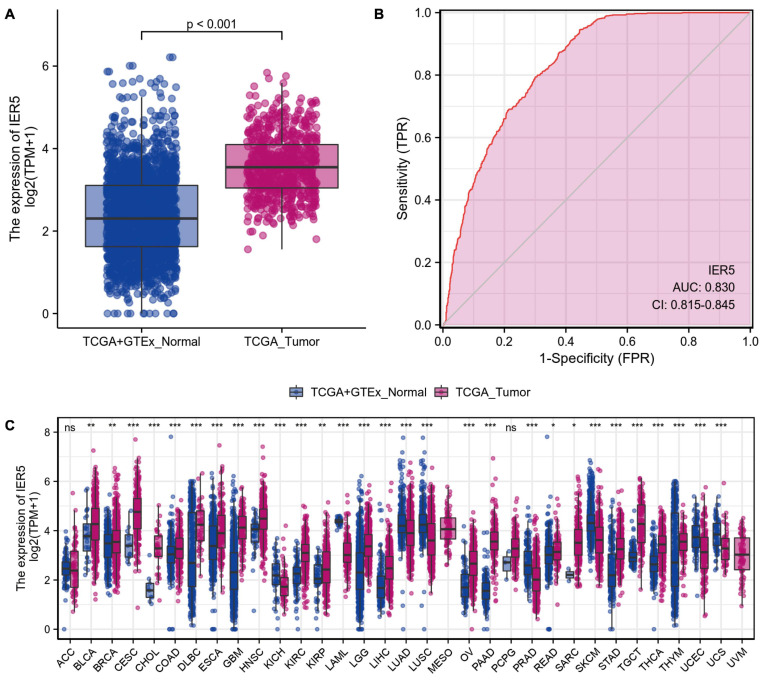
The difference in *IER5* expression levels between tumor and normal tissues. **(A)** Expression levels of *IER5* in glioma and normal samples. **(B)** ROC analysis of *IER5* expression showing promising discrimination power between glioma and non-tumor tissues. **(C)** Pancancer analysis of *IER5* expression across cancers from TCGA. ns, no significance, *P* < 0.05; *, *P* < 0.05; **, *P* < 0.01; ***, *P* < 0.001.

**FIGURE 2:**
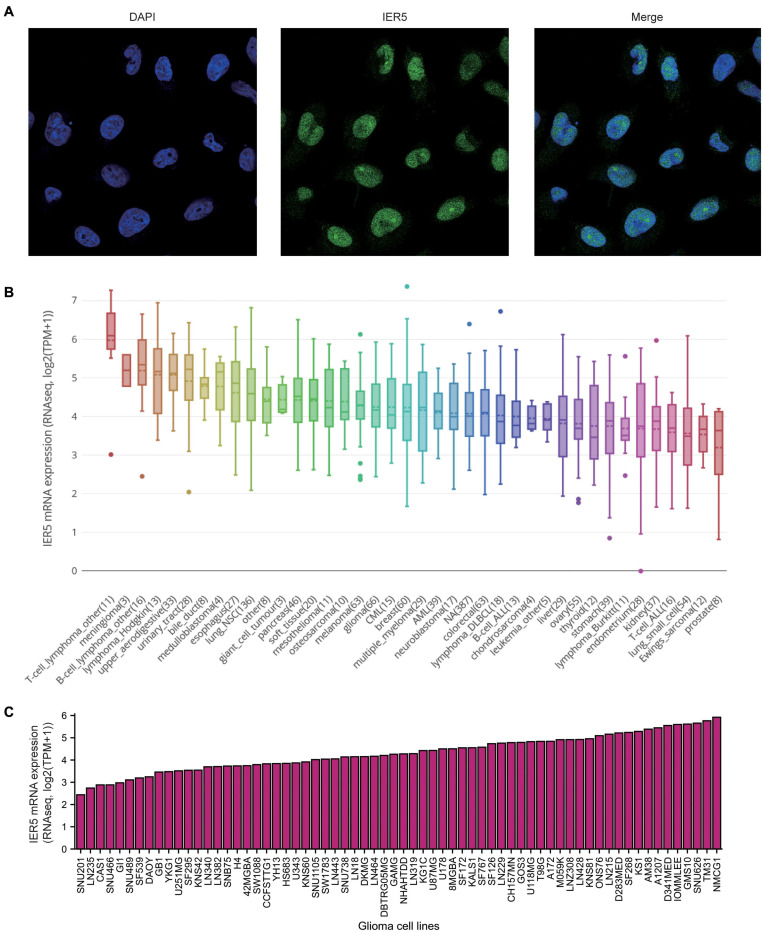
Expression of IER5 at protein levels in U251 cell lines and at mRNA levels in different glioma cell lines. **(A)** Gene expression of IER5 was identified using immunofluorescence in U251 cell lines, these data are from The Human Protein Atlas databases. **(B)** IER5 expression patterns in 1457 cell lines representing 40 distinct tumor types, these data are from The Cancer Cell Line Encyclopedia (CCLE) databases. **(C)** IER5 expression patterns in 66 glioma cell lines, these data are from CCLE databases.

**FIGURE 3:**
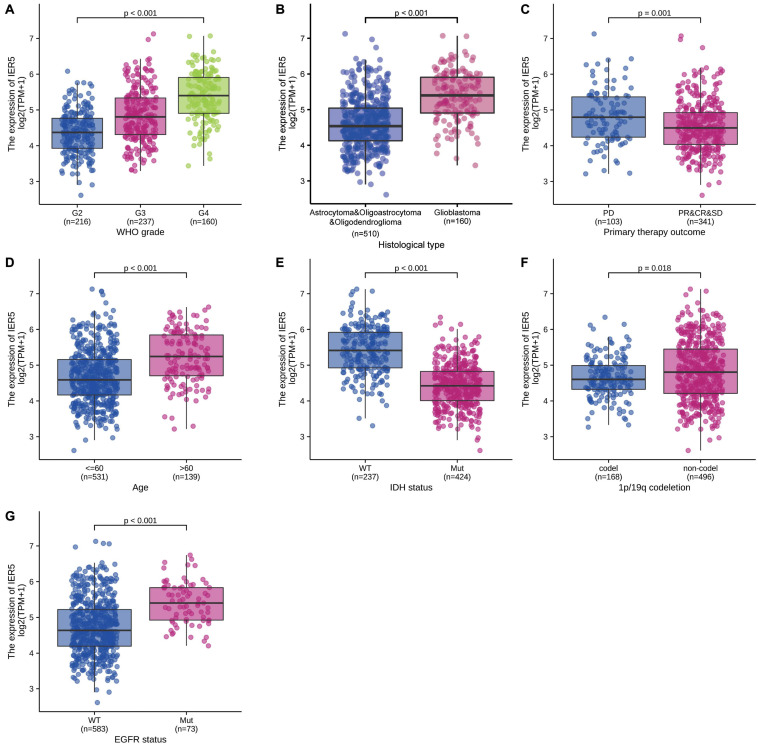
Association of *IER5* expression and clinicopathologic characteristics. **(A)** WHO grade, **(B)** Histological type, **(C)** Primary therapy outcome, **(D)** Age, **(E)** IDH status, **(F)** 1p/19q codeletion, and **(G)** EGFR status.

As shown in Figure 1, we used Wilcoxon signed rank tests to analyze the expression level of *IER5* in tumor and non-malignant tissues. *IER5* expression showed promising discrimination value, and the AUC (area under the ROC curve) value of *IER5* expression for discriminating tumors from healthy tissues was 0.830 (Figure 1B). The results showed that there was remarkably higher expression of *IER5* in tumor tissues in contrast with healthy tissues. In the pan-cancer analysis (Figure 1C), the results illustrated that the expression of *IER5* were elevated in many kinds of tumors. In addition, we also analyzed the expression of *IER5* in cancer cell lines. Immunocytochemical analysis reveled that IER5 fluorescence was mainly distributed in the nucleus in U251 cell lines (Figure 2A). Moreover, the expression of IER5 mRNA in different cancer cell lines and glioma cell lines are showed in Figures 2B,C. All these results indicate that the expression of IER5 is significantly up-regulated in glioma. As shown in Figure 3, the Wilcoxon rank sum test along with the Kruskal–Wallis rank sum test illustrated that the level of *IER5* was dramatically associated with WHO grade (*P* < 0.001), EGFR status (*P* < 0.001), IDH status (*P* < 0.001), primary therapy outcome (*P* = 0.018), histological type (*P* < 0.001), 1p/19q codeletion (*P* = 0.002), and age (*P* < 0.001).

Univariate logistic regression analysis of *IER5* expression illustrated that higher expression levels were significantly linked to more advanced WHO grade [OR = 1.05 (1.04–1.06) for G4 vs. G2 and G3, *P* < 0.001], IDH wild-type (WT) status [OR = 1.11 (1.09–1.13) for WT vs. Mut, *P* < 0.001], 1p/19q codeletion [OR = 0.98 (0.97–0.99) for codeletion vs. non-codeletion, *P* < 0.001], a primary therapy outcome of complete response (CR) [OR = 0.98 (0.97–1.00) for CR vs. progressive disease (PD), stable disease (SD), and partial response (PR), *P* = 0.014], EGFR mutation [OR = 1.03 (1.02–1.04) for Mut vs. WT, *P* < 0.001], and age ≤ 60 years [OR = 0.97 (0.96–0.98) for ≤ 60 vs. >60, *P* < 0.001]. Additionally, the associations between *IER5* expression and clinicopathologic features were confirmed by Chi-square analysis (Table 2). These findings indicate that cancers with a high *IER5* expression level are correlated with poor clinicopathological factors.

**TABLE 2:** *IER5* expression associated with clinical pathological characteristics (logistic regression).

Characteristics	Total (*N*)	Odds ratio (OR)	*P* value
WHO grade (G4 vs. G2 and G3)	613	6.71 (4.34–10.68)	< 0.001
IDH status (WT vs. Mut)	661	13.35 (8.92–20.46)	< 0.001
1p/19q codeletion (codel vs. non-codel)	664	0.56 (0.39–0.80)	0.002
Primary therapy outcome (CR vs. PD, SD, and PR)	444	0.86 (0.56–1.30)	0.471
EGFR status (Mut vs. WT)	656	8.42 (4.32–18.43)	< 0.001
PIK3CA status (Mut vs. WT)	656	1.61 (0.89–2.96)	0.120
Age (≤ 60 vs. >60)	670	0.27 (0.18–0.41)	< 0.001
Gender (Female vs. Male)	670	1.02 (0.75–1.39)	0.876
Histological type (Glioblastoma vs. Astrocytoma, Oligoastrocytoma, and Oligodendroglioma)	670	7.13 (4.63–11.31)	< 0.001

### Survival Outcomes and Multivariate Analysis

**FIGURE 4:**
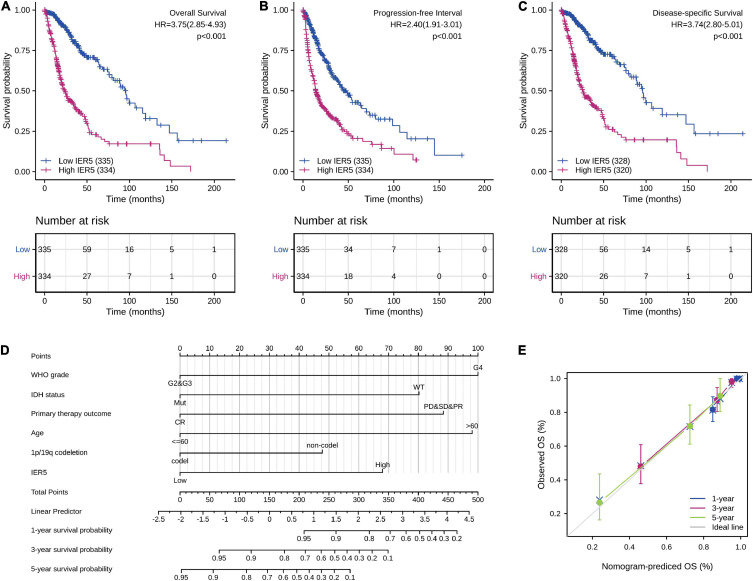
Impact of *IER5* expression on overall survival, progression-free interval, and disease-specific survival in glioma patients in the TCGA cohort **(A–C)**. Construction and performance validation of the *IER5*-based nomogram for glioma patients **(D,E)**.

As indicated in Figure 4, Kaplan–Meier survival assessment showed that glioma individuals with high *IER5* expression had a poorer prognosis in contrast with those with low *IER5* expression (*P* < 0.001). The univariate analysis illustrated that high *IER5* expression was linked to a short OS [hazard ratio (HR): 3.75; 95% CI: 2.85–4.93; and *P* < 0.001], poor progression-free interval (HR: 2.40; 95% CI: 1.91–3.01; and *P* < 0.001), and poor disease-specific survival (DSS; HR: 3.74; 95% CI: 2.80–5.01; and *P* < 0.001). The multivariate analysis revealed that *IER5* expression remained independently correlated with OS, with an HR of 2.474 (95% CI: 1.552–3.943, *P* < 0.001), as did WHO grade, IDH status, 1p/19q codeletion, primary therapy outcome, and age (Table 3).

**TABLE 3:** **(A)** Association with overall survival and clinicopathologic characteristics in glioma patients using Cox regression. **(B)** Multivariate survival model after variable selection.

Characteristics	Total (*N*)	HR (95% CI) univariate analysis	*P* value univariate analysis	HR (95% CI) multivariate analysis	*P* value multivariate analysis
WHO grade (G4 vs. G2 and G3)	612	9.504 (7.162–12.611)	< 0.001	3.928 (1.139–13.538)	0.030
IDH status (WT vs. Mut)	660	9.850 (7.428–13.061)	< 0.001	2.691 (1.488–4.866)	0.001
1p/19q codeletion (codel vs. non-codel)	663	0.216 (0.138–0.338)	< 0.001	0.536 (0.298–0.962)	0.037
Primary therapy outcome (CR vs. PD, SD, and PR)	443	0.238 (0.115–0.489)	< 0.001	0.273 (0.124–0.600)	0.001
Gender (Male vs. Female)	669	1.230 (0.955–1.585)	0.109		
Age (>60 vs. ≤ 60)	669	4.716 (3.609–6.161)	< 0.001	3.540 (2.130–5.885)	< 0.001
Race (White vs. Asian and Black or African American)	657	0.806 (0.492–1.321)	0.393		
EGFR status (Mut vs. WT)	655	3.628 (2.672–4.927)	< 0.001	1.606 (0.786–3.282)	0.194
PIK3CA status (Mut vs. WT)	655	1.011 (0.625–1.635)	0.966		
IER5 (High vs. Low)	669	3.747 (2.847–4.933)	< 0.001	2.474 (1.552–3.943)	< 0.001

### High *IER5* Expression Levels Impact the Prognosis of Glioma in Patients With Different Clinicopathological Features

**FIGURE 5:**
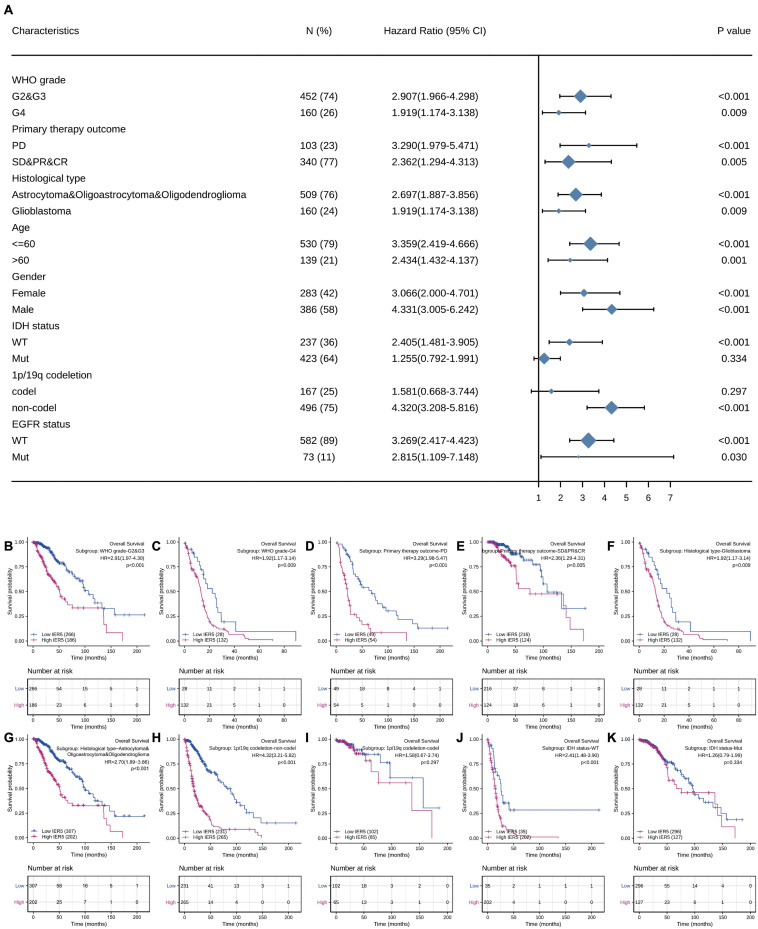
Correlation of *IER5* expression and clinical prognosis in glioma patients with different clinicopathological factors. **(A)** Forest plots showing subgroup analyses of overall survival. **(B–K)** Kaplan–Meier survival subgroup analysis in patients stratified by clinical characteristics.

We used univariate Cox analysis to assess the relationship of *IER5* expression with the clinicopathological characteristics of glioma patients (Figure 5). Elevated expression of *IER5* was correlated with worse OS in female and male patients and patients with different WHO grades, ages, and EGFR statuses. Overexpression of *IER5* was linked to worse OS in WT IDH status patients [HR = 2.405 (1.481–3.905), *P* < 0.001], patients without 1p/19q codeletion [HR = 4.320 (3.208–5.816), *P* < 0.001], patients who had a primary therapy outcome of PD [HR = 3.290 (1.979–5.471), *P* < 0.001], patients with astrocytoma [HR = 5.173 (2.905–9.211), *P* < 0.001], patients with glioblastoma [HR = 1.919 (1.174–3.138), *P* = 0.009], and WT PIK3CA status patients [HR = 3.738 (2.799–4.993), *P* < 0.001]. These correlations were not observed in other patient subgroups. These data indicate that the *IER5* expression level can affect the prognosis of glioma patients with different clinicopathological characteristics.

### Development and Verification of a Nomogram on the Basis of *IER5* Expression and Clinicopathological Factors

To predict the prognosis of glioma patients, we constructed a nomogram that integrated *IER5* expression and independent clinical risk factors (WHO grade, IDH status, primary therapy outcome, age, and 1p/19q codeletion; Figure 4D). The selection of clinical risk factors was according to the multivariate Cox analysis data. In the nomogram, a higher total number of points predicted a worse prognosis than a lower total number of points. We used the C-index and a calibration plot to explore the performance of the prediction model. The C-index for the nomogram was 0.841 (95% CI: 0.822–0.861) with 1,000 bootstrap replicates. The calibration plot of the survival probability at 1, 3, and 5 years illustrated good consistency between the predictions made by the nomogram and the actual observations (Figure 4E). In summary, these data indicate that our nomogram was a better model for estimating the survival probability at 1, 3, and 5 years in glioma patients than independent prognostic factors.

### Validation of the Prognostic Value of *IER5* in TMA and CGGA Datasets

**FIGURE 6:**
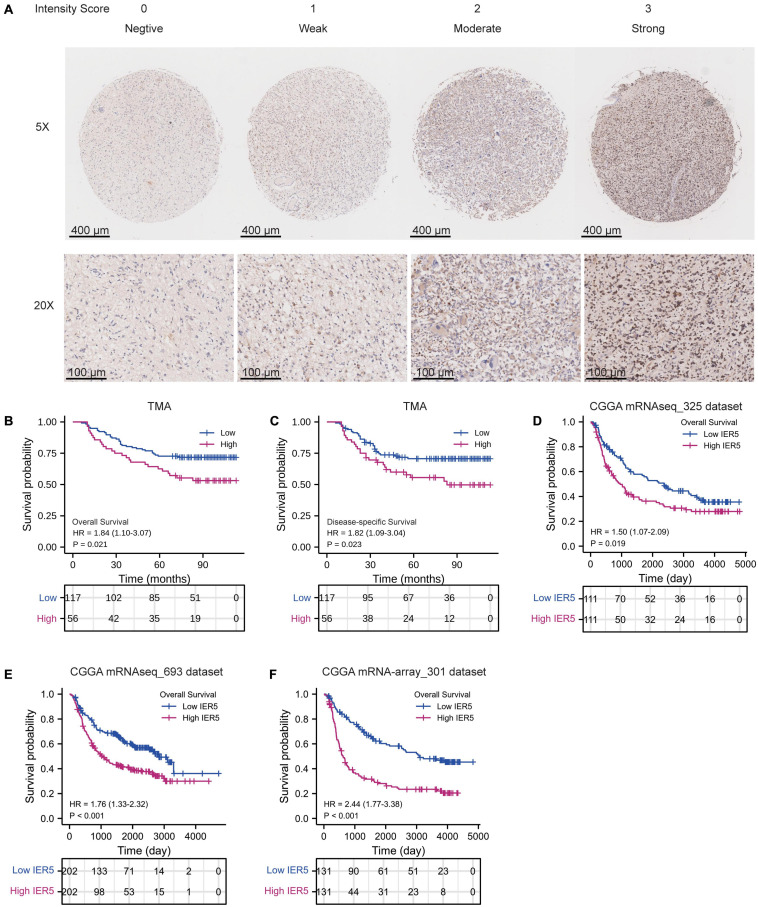
Immunohistochemistry of IER5 expression in the glioma tissue microarray cohort and the validation of prognosis value of *IER5* expression in Chinese Glioma Genome Atlas (CGGA) datasets. **(A)** Representative images of IER5 staining in the negative, weak, moderate, and strong expression groups. **(B)** Kaplan–Meier survival curves of overall survival stratified by IER5 protein expression. **(C)** Kaplan–Meier survival curves of disease-specific survival stratified by IER5 protein expression. **(D–F)** Kaplan–Meier survival curves of overall survival stratified by *IER5* expression in three independent datasets of CGGA.

Using TMA containing samples from 180 glioma patients, the protein expression levels of *IER5* were analyzed through IHC staining. In Figure 6A, the representative negative, weak, moderate, and strong staining of IER5 in glioma tissues were illustrated (Table 4). Kaplan–Meier analysis illustrated that high *IER5* protein expression was significantly related to shorter OS (HR: 1.84; 95% CI: 1.10–3.07; *P* = 0.021; Figure 6C) and poorer DSS (HR: 1.82; 95% CI: 1.09–3.04; *P* = 0.023; Figure 5C) than low *IER5* protein expression. In addition, we also use CGGA to validate the prognostic value of *IER5* in three independent datasets (including mRNAseq_325, mRNAseq_693, and mRNA-array_301). Kaplan–Meier analysis based on CGGA showed that high *IER5* expression was significantly correlated to shorter OS in mRNAseq_325 dataset (HR: 1.50; 95% CI: 1.07–2.09; *P* = 0.019; Figure 6C), mRNAseq_693 dataset (HR: 1.76; 95% CI: 1.33–2.32; *P* < 0.001; Figure 6E), and mRNA-array_301 dataset (HR: 2.44; 95% CI: 1.77–3.38; *P* < 0.001; Figure 6F). These results confirmed that the *IER5* expression was able of prediction the prognosis of glioma patients.

**TABLE 4:** Correlation of *IER5* expression and clinical prognosis in glioma patients with different clinicopathological characteristics.

Characteristics	*N* (%)	HR (95% CI)	*P* value
**WHO grade**			
G2 and G3	452 (74)	2.907 (1.966–4.298)	< 0.001
G4	160 (26)	1.919 (1.174–3.138)	0.009
**Primary therapy outcome**			
PD	103 (23)	3.290 (1.979–5.471)	< 0.001
SD, PR, and CR	340 (77)	2.362 (1.294–4.313)	0.005
**Histological type**			
Astrocytoma, Oligoastrocytoma, and Oligodendroglioma	509 (76)	2.697 (1.887–3.856)	< 0.001
Glioblastoma	160 (24)	1.919 (1.174–3.138)	0.009
**Age**			
≤ 60	530 (79)	3.359 (2.419–4.666)	< 0.001
>60	139 (21)	2.434 (1.432–4.137)	0.001
**Gender**			
Female	283 (42)	3.066 (2.000–4.701)	< 0.001
Male	386 (58)	4.331 (3.005–6.242)	< 0.001
**IDH status**			
WT	237 (36)	2.405 (1.481–3.905)	< 0.001
Mut	423 (64)	1.255 (0.792–1.991)	0.334
**1p/19q codeletion**			
Codel	167 (25)	1.581 (0.668–3.744)	0.297
Non-codel	496 (75)	4.320 (3.208–5.816)	< 0.001
**EGFR status**			
WT	582 (89)	3.269 (2.417–4.423)	< 0.001
Mut	73 (11)	2.815 (1.109–7.148)	0.030

### Identification of DEGs Between Glioma Patients With High- and Low-*IER5* Expression

**FIGURE 7:**
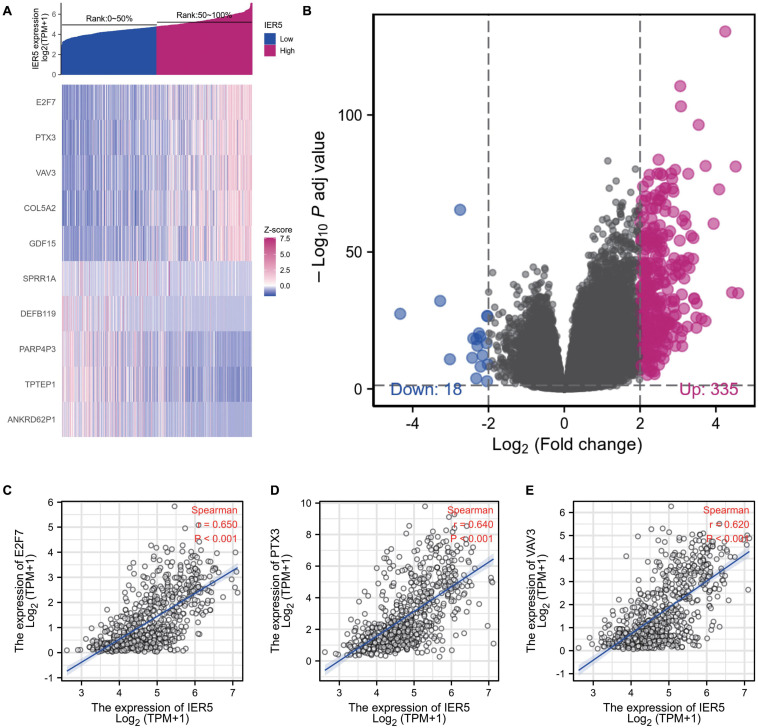
DEGs between glioma patients with high- and low- *IER5* expression. **(A)** Heat map of the 10 differentially expressed genes in the high- and low-*IER5* expression groups. **(B)** Volcano plot of differentially expressed genes. **(C–E)** Correlation of *IER5* expression with expression of *E2F7* **(C)**, *PTX3* **(D)**, and *VAV3* **(E)** in the scatter plot.

The HTSeq-Counts data from TCGA were analyzed using the R package DESeq2 (with threshold values of adjusted *P* < 0.05 along with |log2 FC| > 2; Love et al., 2014). DEG expression was visualized with a heat map and volcano plot (Figures 7A, B). As shown in the heat map, the top 5 gene sets were significantly positively and negatively correlated with *IER5* (Figure 7A). As shown in the volcano plot, there were 335 genes with a significant positive correlation with *IER5* and 18 genes with a significant negative correlation with *IER5* (Figure 7B). Scatter plots of individual genes showed a significant positive correlation between *IER5* and *E2F7* (Spearman *r* value = 0.650, *P*-value < 0.001), *PTX3* (Spearman *r* value = 0.640, *P*-value < 0.001), and *VAV3* (Spearman *r* value = 0.620, *P*-value < 0.001) expression (Figures 7C–E). *E2F7*, *PTX3*, and *VAV3* have been reported as oncogene in many kinds of tumors especially in glioma (Liu et al., 2014, 2018; Yang et al., 2020). These results illuminated that *IER5* may have a wide range of functions through modulating different genes.

### Functional Enrichment Analysis of DEGs

**FIGURE 8:**
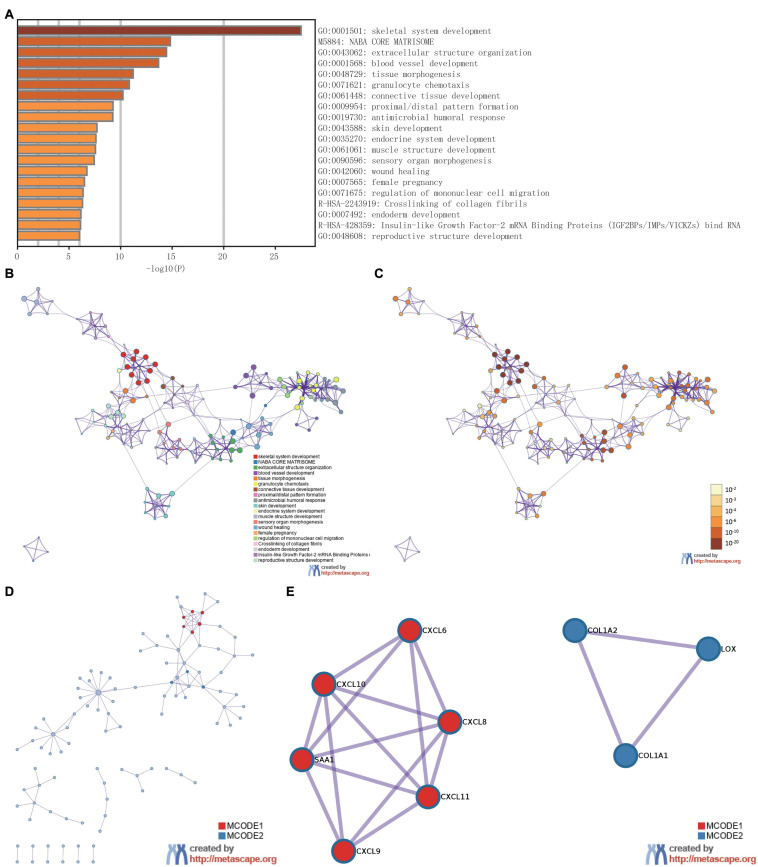
The enrichment analysis and visualized protein-protein interaction (PPI) enrichment analysis of DEGs (Metascape). **(A)** A heatmap of Gene Ontology analysis colored by *P*-values. **(B,C)** An interactive network of the top 20 enrichment terms. **(D,E)** PPI network and densely connected network components identified by the Molecular Complex Detection (MCODE) algorithm.

To analyze the functional implication information of *IER5* in glioma patients, GO, and KEGG functional enrichment assessments were performed within Metascape using the 353 DEGs identified between the high- and low-*IER5* expression groups. *IER5-*associated genes were found to be linked to several biological processes, cellular components, and molecular functions. We found that skeletal system development, extracellular structure organization, blood vessel development, tissue morphogenesis, granulocyte chemotaxis, connective tissue development, skin development, endocrine system development, and muscle structure development were related to the regulation of *IER5-*related genes (Figure 8). Overall, the DEGs were closely associated with embryonic development. In addition, we analyzed the PPI network by Metascape to better understand the role of *IER5* in the development of glioma (Figure 8D). The significant densely connected network constituents were shown in Figure 8E, and each network is assigned a unique color.

### Gene-Related Signaling Pathways Identified by GSEA

**FIGURE 9:**
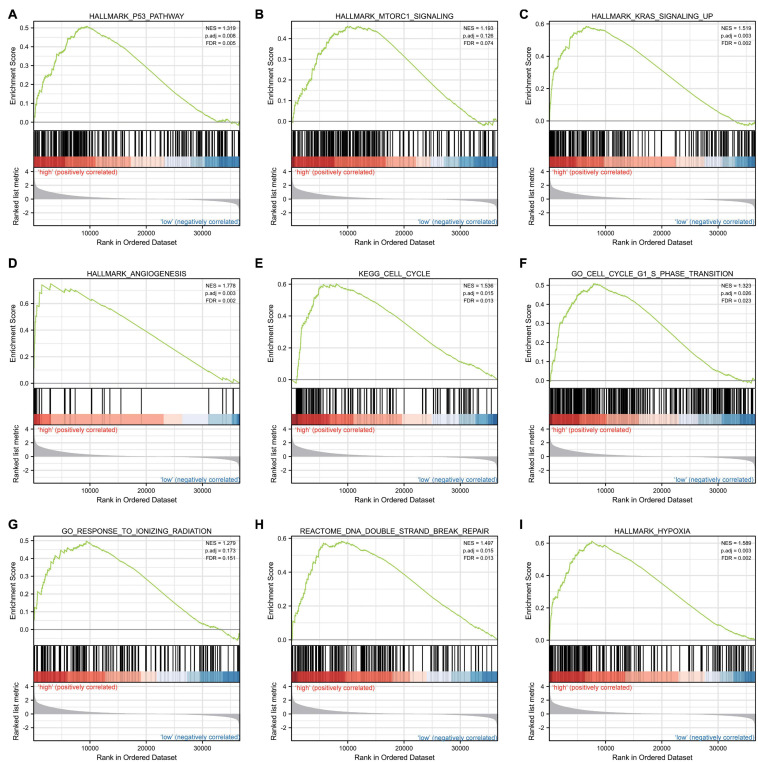
Enrichment plots from gene set enrichment analysis (GSEA). Several pathways and biological processes were enriched, including the **(A)** p53 pathway, **(B)** MTORC1 signaling, **(C)** KRAS signaling, **(D)** angiogenesis, **(E)** cell cycle, **(F)** cell cycle G1 S phase transition, **(G)** response to ionizing radiation, **(H)** DNA double strand repair and **(I)** hypoxia.

Gene set enrichment analysis was carried out to determine the biological signaling cascades related to glioma by comparing the high- and low-*IER5* expression datasets. Dramatic differences (FDR < 0.25, adjusted *P-*value < 0.05) in the enrichment of many pathways in the MSigDB Collection (c2.cp.v7.2.symbols, h.all.v7.2.symbols, and c5.all.v7.2.symbols) were revealed. Figure 9 shows the PPI network of *IER5* and its potential co-expression genes within the *IER5*-related DEGs. Nine pathways, including pathways related to P53, MTORC1 signaling, KRAS signaling, angiogenesis, the cell cycle, the cell cycle G1 S phase transition, the response to ionizing radiation, DNA double-strand repair and hypoxia, exhibited remarkably differential enrichment in the *IER5-*high expression phenotype, indicating the potential role of *IER5* in the onset of glioma.

### Correlation Between *IER5* Expression and Immune Infiltration

**FIGURE 10:**
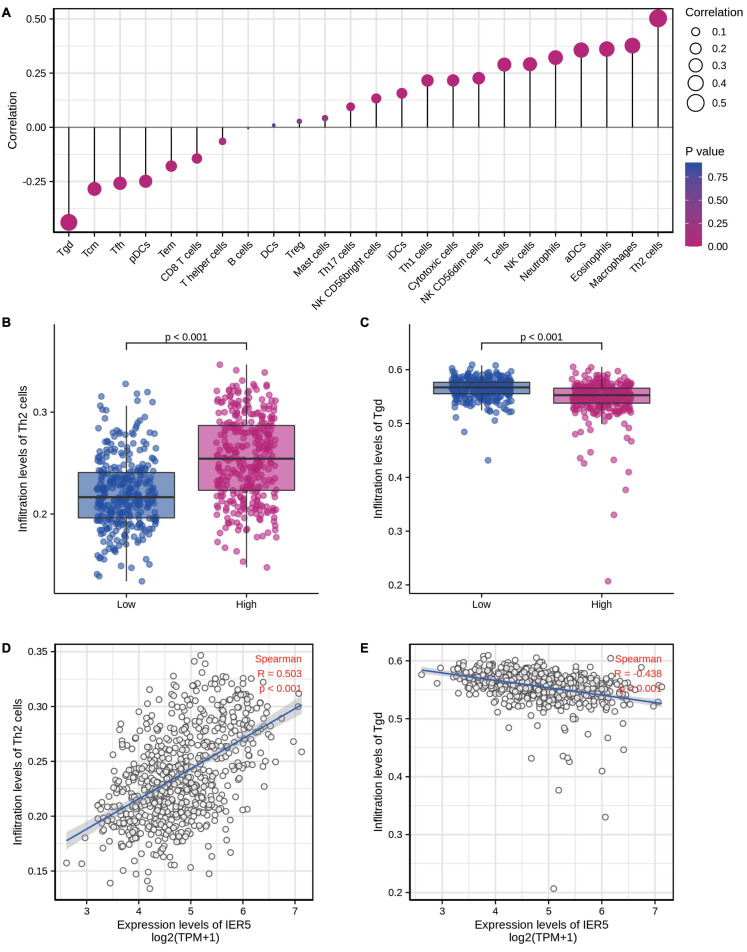
The expression level of *IER5* was associated with immune infiltration in the tumor microenvironment. **(A)** Correlation between the relative abundances of 24 immune cells and *IER5* expression levels. **(B,C)** Infiltration levels of Th2 cells and Tgd cells in the low- and high-*IER5* expression groups. **(D,E)** Correlation between the relative enrichment score of Th2 cells and Tgd cells and the expression level of *IER5.*

We used Spearman correlation analysis to determine the association between the expression level (in TPM values) of *IER5* and the immune cell invasion level (quantified by ssGSEA). As indicated in Figure 10, T helper 2 (Th2) cells were remarkably positively linked to *IER5* expression, with a Spearman *r* value of 0.503 and a *P*-value < 0.001. Additionally, in contrast with the *IER5*-low expression group, the *IER5*-high expression group had a significantly higher enrichment score of Th2 cells as per the Wilcoxon rank sum test. The levels of other immune cells, including macrophages, eosinophils, activated dendritic cells (aDCs), and gamma delta (γδ) T cells, were moderately correlated with *IER5* expression.

## Discussion

Previous investigations have reported the expression, as well as the functions of *IER5* in cancers (Asano et al., 2016; Yang et al., 2016). Growth-promoting stimuli and genotoxic factors such as radiation can activate *IER5* expression (Ishikawa and Sakurai, 2015; Shi et al., 2016). *IER5* may influence cell proliferation along with survival in response to ionizing radiation and thermal stress by regulating cell cycle checkpoints (Ding et al., 2009, 2019). Nonetheless, the expression level of *IER5* and its prognostic significance in glioma patients remain unclear.

Herein, bioinformatics analyses of the prognostic significance of *IER5* expression in glioma patients were carried out using high-throughput RNA-sequencing data from TCGA. *IER5* overexpression in glioma patients was correlated with advanced clinicopathological characteristics (advanced WHO grade, WT IDH status, a primary therapy outcome of CR, age ≤ 60 years, and 1p/19q codeletion), short survival time, as well as poor prognosis. We also found that *IER5* expression was associated with poor prognosis in CGGA datasets. Moreover, we conducted IHC using a TMA to assess the association of the protein level of IER5 with the prognosis of individuals with glioma. The IHC results illustrated that individuals with high IER5 protein levels had poor prognosis. This result was consistent with our bioinformatic results. To elucidate the function of *IER5* in glioma patients, we performed GSEA and found that pathways related to p53, MTORC1 signaling, angiogenesis, apoptosis, the cell cycle, KRAS signaling, and response to ionizing radiation were enriched differentially in the *IER5*-high expression phenotype. All the results suggest that the potential of *IER5* as a prognostic biomarker and treatment target in glioma patients. In the following sections, we will discuss some critical points of our study.

We performed Kaplan-Meier survival assessment to explore the utility of *IER5* expression levels in glioma patients stratified by clinicopathological characteristics. High *IER5* contents were linked to poor prognosis in the subgroup analyses. These subgroups included groups categorized according to WHO grade, primary therapy outcome, age, gender, IDH status, histological type, 1p/19q codeletion, and EGFR status. The results strongly suggest that *IER5* is a powerful prognostic biomarker within these subsets. It is interesting that the Kaplan–Meier survival analysis illustrated a remarkable association of *IER5* expression level with OS in WT IDH status gliomas but not mutant gliomas in patients with 1p/19q codeletion gliomas but not in patients without 1p/19q codeletion. These results indicate that the relationship of *IER5* expression level with survival may be influenced by IDH status, as well as 1p/19q codeletion.

We developed a nomogram to predict the survival of glioma patients. Our findings illustrate that *IER5* is an independent prognostic marker for glioma. The nomogram included WHO grade, IDH status, primary therapy outcome, age, 1p/19q codeletion and *IER5* expression. A previous study reported that age is an independent predictor of glioma prognosis, and an older age conferred a worse prognosis (Ostrom et al., 2014a). IDH mutation is an early event in gliomagenesis and prevents glioma progression (Weller et al., 2015). The codeletion of chromosome arms 1p, as well as 19q (1p/19q codeletion) is linked to glioma sensitivity to chemotherapy and the astrocytic histologic type (Eckel-Passow et al., 2015). Tumors without 1p/19q codeletion, WT IDH, PD as the primary therapy outcome and high WHO grade (III or IV) tend to have a poor outcome (Weller et al., 2015; Kristensen et al., 2019). Our results are consistent with those published in the literature. Based on the calibration plot, the actual values for 1, 3, and 5 year OS were in good agreement with the predicted values. Therefore, our nomogram could be a potential novel prognostic strategy to be used in the clinic in the future.

Previous studies have documented that *IER5* may be employed as a molecular biomarker for biodosimetry purposes upon ionizing radiation exposure (Yu et al., 2017). Several studies have reported the functions of *IER5* in the response to ionizing radiation and modulation of the cell cycle (Ding et al., 2009; Nakamura et al., 2011). The activation of *IER5* after radiation may promote cell survival by modulating the cell cycle. In this study, we similarly found that *IER5* was associated with positive modulation of the cell cycle and the G1/S phase transition and the response to ionizing radiation. Studies have reported that p53 activates *IER5* transcription by localizing in the vicinity of its promoter upon DNA damage. The activation of *IER5* can lead to tumor progression (Toma-Jonik et al., 2019). Herein, there was enrichment of the p53 cascade in the *IER5*-high expression phenotype. Besides, we established that high *IER5* expression was linked to the KRAS signaling pathway, MTORC1 signaling pathway, hypoxia and angiogenesis by GSEA. These pathways and pathological processes are related to glioblastoma pathogenesis (Pachow et al., 2015; Boyd et al., 2021; Hajj et al., 2021). However, this is the first report of the association of *IER5* expression with the KRAS signaling pathway, MTORC1 signaling pathway, hypoxia and angiogenesis. The precise regulatory mechanisms responsible for these associates remain incompletely understood and need to be further studied.

Another equally pivotal aspect of this study is the finding that *IER5* expression is linked to the invading levels of diverse immune cells in glioma. We found that the associations of *IER5* with Th2 cells and γδ T cells were the strongest. Our results show that the high *IER5* expression group has increased levels of Th2 cell, whereas the levels of γδ T cells were decreased. Our results suggest possible mechanisms by which *IER5* regulates the infiltration of Th2 cells, along with γδ T cells in glioma patients. Th2 cytokines such as IL-4, IL-10, IL-5, IL-9, and IL-6 can down-regulate tumor-specific immunity (Protti and De Monte, 2020). These cytokines along with their receptors were highly expressed in glioblastoma samples and cell lines (Hao et al., 2002). The overexpression of Th2 cytokines and increased Th1 infiltration in glioblastoma are associated with poor prognosis (Hao et al., 2002; Gousias et al., 2010; Piperi et al., 2011). γδ T cells paly vital roles in antitumor immunity and the ability to kill tumor cells (Lee et al., 2019). In glioblastoma patients, γδ T cell proliferation is impaired, and γδ T cell deficiency occurs in the tumor microenvironment (Bryant et al., 2009). We speculate that overexpression of *IER5* promotes Th2 cell infiltration and induces γδ T cell depletion. This situation indicates an “immunosuppressive state” in glioblastoma and could account for the poor therapeutic effect of immunotherapy in such tumors. In summary, *IER5* likely plays a core role in the modulation of immune cell infiltration in glioma. However, to understand the precise regulatory relationships between *IER5* and immune cells, more preclinical and clinical data are needed.

Although our study revealed the association of *IER5* with glioma, there were still some limitations that remain to be addressed. First, most of our findings were obtained from bioinformatics analysis and public databases, which lack experimental verification in cells. Second, our results were mainly based on the RNA sequencing data from TCGA data resource. Data on the expression levels of proteins other than IER5 in patients were lacking, and we could not explore the direct mechanism of IER5 in the development of glioma. Therefore, we will perform laboratory experiments to further validate our results and investigate the mechanism of *IER5* in glioma.

In conclusion, the *IER5* expression might serve as a reliable molecular marker for patient survival in glioma. Moreover, pathways related to the cell cycle, the P53 pathway, MTORC1 signaling, KRAS signaling, angiogenesis and response to ionizing radiation may be the key pathways regulated by *IER5* in glioma patients. This study may be beneficial for elucidating the clinicopathological significance and molecular underpinning of glioma. However, further studies should be conducted to validate the molecular mechanism and the clinical application of *IER5* as a prognostic indicator or therapeutic target for glioma patients.

## Data Availability Statement

The original contributions presented in the study are included in the article/supplementary material, further inquiries can be directed to the corresponding author/s.

## Author Contributions

MW and SL contributed to the conception of the study. ZW and DW performed the data analyses and wrote the manuscript. FZ, YZ, GZ, YM, and BS helped perform the analysis with constructive discussions. All authors contributed to the article and approved the submitted version.

## Conflict of Interest

The authors declare that the research was conducted in the absence of any commercial or financial relationships that could be construed as a potential conflict of interest.

## Footnotes

1 http://www.cgga.org.cn/index.jsp

2 http://metascape.org

3 http://www.bioconductor.org/packages/release/bioc/html/GSVA.html

4 https://www.egene.org.cn/cms/g-index.jhtml
